# A comparison of random forests, boosting and support vector machines for genomic selection

**DOI:** 10.1186/1753-6561-5-S3-S11

**Published:** 2011-05-27

**Authors:** Joseph O Ogutu, Hans-Peter Piepho, Torben Schulz-Streeck

**Affiliations:** 1 Bioinformatics Unit, Institute of Crop Science, University of Hohenheim, Fruwirthstrasse 23, 70599 Stuttgart, Germany

## Abstract

**Background:**

Genomic selection (GS) involves estimating breeding values using molecular markers spanning the entire genome. Accurate prediction of genomic breeding values (GEBVs) presents a central challenge to contemporary plant and animal breeders. The existence of a wide array of marker-based approaches for predicting breeding values makes it essential to evaluate and compare their relative predictive performances to identify approaches able to accurately predict breeding values. We evaluated the predictive accuracy of random forests (RF), stochastic gradient boosting (boosting) and support vector machines (SVMs) for predicting genomic breeding values using dense SNP markers and explored the utility of RF for ranking the predictive importance of markers for pre-screening markers or discovering chromosomal locations of QTLs.

**Methods:**

We predicted GEBVs for one quantitative trait in a dataset simulated for the QTLMAS 2010 workshop. Predictive accuracy was measured as the Pearson correlation between GEBVs and observed values using 5-fold cross-validation and between predicted and true breeding values. The importance of each marker was ranked using RF and plotted against the position of the marker and associated QTLs on one of five simulated chromosomes.

**Results:**

The correlations between the predicted and true breeding values were 0.547 for boosting, 0.497 for SVMs, and 0.483 for RF, indicating better performance for boosting than for SVMs and RF.

**Conclusions:**

Accuracy was highest for boosting, intermediate for SVMs and lowest for RF but differed little among the three methods and relative to ridge regression BLUP (RR-BLUP).

## Background

Genomic selection is a method for estimating GEBVs using dense molecular markers spanning the entire genome [1]. Given the wide range of approaches for predicting GEBVs, it is important to evaluate their performance, pros and cons to identify those able to accurately predict GEBVs. Here, we compare predictive performances among three of the most powerful machine learning methods with demonstrated high predictive accuracies in many application domains, namely RF [2,3]; boosting [5] and SVMs [5,6] and with RR-BLUP [7] for predicting breeding values for quantitative traits.

RF has several appealing properties that make it potentially attractive for GS [2,4]: (*i*) the number of markers can far exceed that of observations, (*ii*) all markers, including those with weak effects, highly correlated and interacting markers have a chance to contribute to the model fit, (*iii*) complex interactions between markers can be easily accommodated, (*iv*) they can perform both simple and complex classification and regression accurately, (*v*) they often require modest fine-tuning of parameters and the default parameterization often performs well [2,3], and (*vi*) they make no distributional assumptions about the predictor variables. Boosting is a stagewise additive model fitting procedure that can enhance the predictive performance of weak learning algorithms [5]. SVMs perform robustified regression using kernel functions of inner products of predictors [5].

We comparatively evaluated the predictive performance of the three machine learning methods and RR-BLUP for estimating GEBVs using the common dataset simulated for the QTLMAS 2010 workshop. RF regression was used to rank the SNPs in terms of their predictive importance.

## Methods

### Data

The simulated data set contained 3226 individuals spanning five generations out of which 2326, constituting the first four generations, were phenotyped and genotyped for 10031 biallelic SNPs arrayed on a genome encompassing five chromosomes. The remaining 900 individuals, representing the fifth generation, had genomic but lacked phenotypic records on the single quantitative trait. The covariate for each genotype with alleles *A*_1_ and *A*_2_ was set to 1 for *A*_1_*A*_1_, -1 for *A*_2_*A*_2_ and 0 for *A*_1_*A*_2_ or*A*_2_*A*_1_.

### Random forests

RF regression uses an ensemble of unpruned decision trees, each grown using a bootstrap sample of the training data, and randomly selected subsets of predictor variables as candidates for splitting tree nodes. The RF regression prediction for a new observation *x* () is made by averaging the output of the ensemble of *B* trees  as [5]:(1)

where Ψ*_b_* characterizes the *b*th RF tree in terms of split variables, cutpoints at each node, and terminal node values.

We implemented RF in the R package *randomForest* with decision trees as base learners [3]. Following various recommendations [2,3], we evaluated different combinations of the values of the number of trees to grow, *ntree* = {500, 1000, 2000}, the number of SNPs randomly selected at each tree node, *mtry* = {0.5, 1, 2} × the default value of *mtry* of sample size/3 for regression, and the minimum size of terminal nodes of trees, below which no split is attempted, *nodesize* = 1. The parameter configuration with the highest prediction accuracy was *ntree* =1000, *mtry* = 3000 and *nodesize* =1. We ranked SNPs by the relative importance of their contributions to predictive accuracy, quantified by how much prediction error increased when the observations left out of the bootstrap samples, the out-of-bag data for a SNP, were randomly permuted while data for all the other SNPs were left unchanged [2,3].

### Stochastic Gradient Boosting

Boosting is an ensemble learning method for improving the predictive performance of classification or regression procedures, such as decision trees [5]. Gradient-boosted models can also handle interactions, automatically select variables, are robust to outliers, missing data and numerous correlated and irrelevant variables and can construct variable importance in exactly the same way as RF [5]. Boosting iteratively adds basis functions in a greedy fashion such that each additional basis function further reduces the selected loss (error) function [5,9]:(2)

where *β_m_*, *m* =1,2,…, *M* are the basis expansion coefficients, and *b*(*x*, *γ*) are simple functions of the multivariate argument *x*, with a set of parameters *γ*=(*γ*_1_,*γ*_2_,…,*γ_M_*).

We used regression trees as basis functions. Boosting regression trees involves generating a sequence of trees, each grown on the residuals of the previous tree [5,9]. Prediction is accomplished by weighting the ensemble outputs of all the regression trees. We used stochastic gradient boosting, assuming the Gaussian distribution for minimizing squared-error loss in the R package *gbm*[9]. We determined the main tuning parameter, the optimal number of iterations (or trees), using an out-of-bag estimate of the improvement in predictive performance. This evaluates the reduction in deviance based on observations not used in selecting the next regression tree. The minimum number of observations in the trees' terminal nodes was set to 1, the shrinkage factor applied to each tree in the expansion to 0.001 and the fraction of the training set observations randomly selected to propose the next tree in the expansion to 0.5. With these settings boosting regression trees with at most 8-way interactions between SNPs required 3656 iterations for the training dataset based on inspecting graphical plots of the out-of-bag change in squared error loss against the number of iterations [9].

### Support Vector Machines (SVMs)

SVMs perform robustified regression for quantitative responses by exploiting the relationships between observations by arraying predictors in observation space using a set of inner products. For regression with a quantitative response, SVM uses the model(3)

where the basis functions, *h*(*x*)*^T^*, which can be linear (or nonlinear) transformations of one (or more) predictors (*x*), are additively combined with the vector of weights (*β*). We used the “*ε*-insensitive” SVM regression that uses only residuals smaller in absolute value than some constant (*ε*) and a linear loss function for larger residuals. This is a robustified regression for which the minimization exercise can be written in regularized sum of squares form [5,6] as:(4)

where(5)

is an “*ε*-insensitive” error measure, ignoring errors less than *ε*, *λ* is a positive constant that controls the trade-off between the approximation error and the amount up to which deviations larger than *ε* are tolerated to get solutions for the SVM regression problem, *y* is a quantitative response and  denotes the norm under a Hilbert space. The SVM optimization procedure produces solution functions of the form [5,6]:(6)

where  are positive weights given to each observation and estimated from the data and the inner product kernel *K*(*x_i_*,*x_j_*) is a *N* × *N* symmetric and positive definite matrix [5]. Typically only a subset of  are nonzero, and the associated observations are called support vectors, hence the name support vector machines. Since the solution depends on the input values only through the inner products *K*(*x_i_*,*x_j_*), a flexible fitting is achieved by transforming the cross-products using the kernel function (*K*(*x_i_*,*x_j_*)) that alters how two observations are related to each other.

We used the *ε*-insensitive SVM regression with a linear kernel to predict GEBVs in the R package *e1071*[8] with an insensitivity zone of *ε* = 10 and a regularization (cost) parameter (*λ* > 0) of *λ* = 0.001 determined by grid search.

### Assessing prediction performance

We used 5-fold cross-validation and the Pearson correlation between the simulated values and predicted GEBVs from the validation set and between the predicted and true breeding values (TBVs) for the non-phenotyped individuals constituting the fifth generation to quantify the predictive accuracy of each method. The training and validation sets respectively contained 60 and 15 crosses and encompassed all phenotyped individuals except the 20 founders.

## Results and discussion

The correlations between the simulated values and predicted GEBVs indicated better performance for boosting and SVMs than for RF (Table 1). The correlations between the predicted and true breeding values (TBVs) for the non-phenotyped individuals were also highest for boosting. These accuracies were comparable with that for RR-BLUP (Table 1). Although boosting and SVMs apparently outperformed RF, SVMs was computationally intensive, especially the grid search for tuning its parameters.

**Table 1:** 

CV/TBV	Sample size		Random Forests		Boosting		Support Vector Machines		Ridge Regression BLUP	
	Mean	Range	Mean	Range	Mean	Range	Mean	Range	Mean	Range

CV	439	416-514	0.466	0.392-0.534	0.503	0.431-0.567	0.503	0.432-0.567	0.530	0.451-0.620

TBV	900		0.483		0.547		0.497		0.607	

Predictive accuracies of random forests, boosted regression trees, epsilon support vector machines and RR-BLUP, expressed as the Pearson correlation between GEBVs and observed values from the 5-fold cross-validation (CV) and between GEBVs and TBV for non-phenotyped individuals (TBV).

-Table 1-

**Figure 1:**
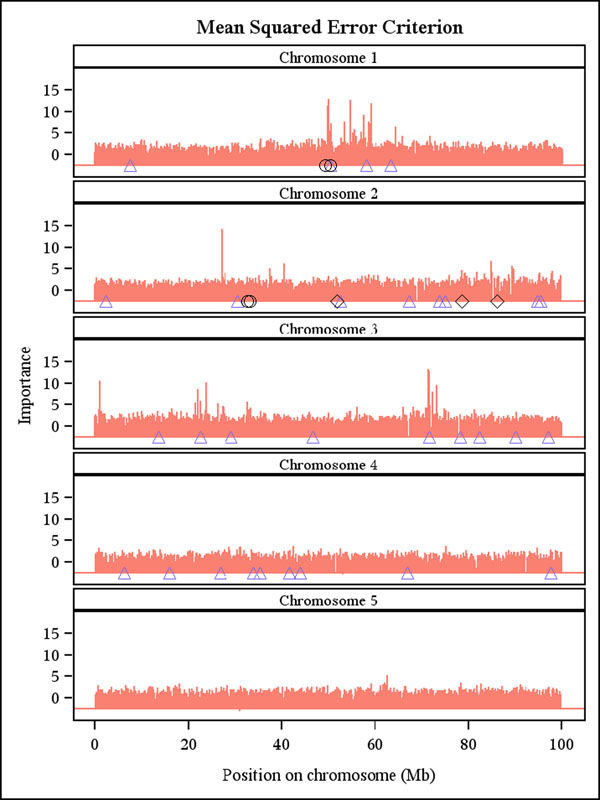
**Importance ranking of the 10031 SNP markers by random forest using percent increase in mean squared error.** Positions of the simulated additive (triangle), epistatic (circle) and imprinted (diamond) QTLs are indicated on each chromosome.

**Figure 2:**
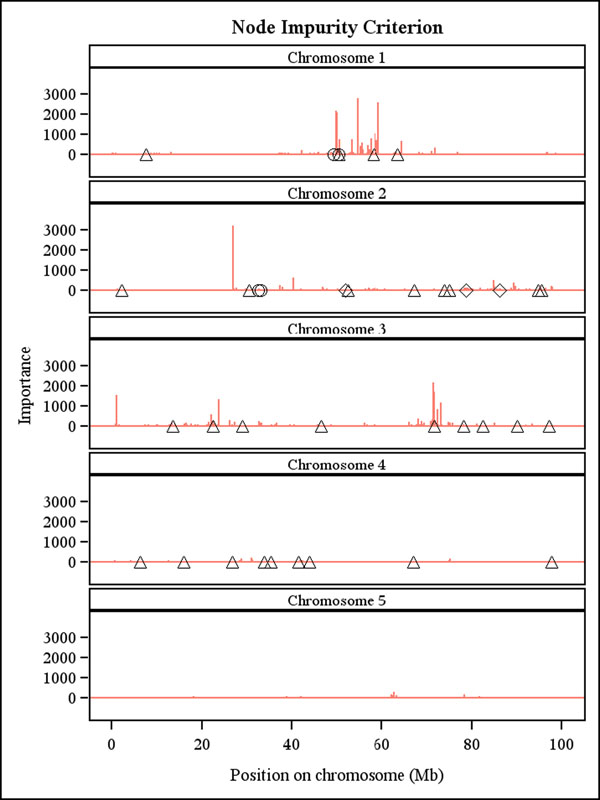
**Importance ranking of the 10031 SNP markers by random forest using tree node impurity.** Positions of the simulated additive (triangle), epistatic (circle) and imprinted (diamond) QTLs are indicated on each chromosome.

RF produced reasonable importance rankings of the SNPs (Figure 1 and Figure 2), which can be used to pre-screen promising markers for further testing.

The two ensemble methods can accommodate complex relationships and interactions (epistasis), which is a potential advantage, but the simulated data did not display many such interactions. A few simulated interacting SNPs with large effects were ranked highly but not top-ranked by RF possibly because RF and boosting had to randomly subsample the 10031 predictors. Thus, it may happen that the SNPs closest to a QTL are not sufficiently frequently sampled, so that the signal of the QTL is captured by other more distant SNPs. Consequently, the signal of a QTL gets blurred relative to classical QTL mapping approaches, which always scan all the markers. This may be one reason that these methods may not perform as well as some other much simpler competitors (e.g., RR-BLUP, BayesB). Nevertheless, for data with complex traits controlled by many genes that show epistatic interactions, the machine learning methods hold much promise and perhaps may even outperform BLUP. Not surprisingly, Moser et al. [10] found the accuracy of SVMs to be the highest among five methods (including BLUP) used to predict GEBVs of dairy bulls from empirical data.

## Conclusions

Predictive accuracies of all three methods were remarkably similar, but boosting and SVMs performed somewhat better than RF. Although boosting was only slightly better than the other methods, it holds perhaps the greatest promise for GS because of its wide versatility, allowing it to assume simpler, faster and more interpretable forms, such as componentwise boosting, able to incorporate automatic predictor selection.

## Competing interests

The authors declare that they have no competing interests.

## Authors’ contributions

JOO conceived the study, conducted the statistical analysis and drafted the manuscript. HPP participated in discussions, helped refine the manuscript and oversaw the project. TSS participated in discussions, data preparation and analysis, and writing of the manuscript. All the authors read and approved the manuscript.
